# Detection of *Aeromonas hydrophila* possessing *aerolysin* gene using gold nanoparticle probe

**DOI:** 10.5455/javar.2023.j714

**Published:** 2023-12-31

**Authors:** Arren Christian M. de Guia, Mary Rose D. Uy-de Guia, Juvy J. Monserate, Joel R. Salazar, Ravelina R. Velasco, Claro N. Mingala, Karl Marx A. Quiazon

**Affiliations:** 1 Livestock Biotechnology Center, Philippine Carabao Center, Science City of Muñoz, The Philippines; 2 Freshwater Aquaculture Center—College of Fisheries, Central Luzon State University, Science City of Muñoz, The Philippines; 3 Production System and Nutrition Section, Philippine Carabao Center, Science City of Muñoz, The Philippines; 4 Department of Chemistry, College of Science, Central Luzon State University, Science City of Muñoz, The Philippines; 5 Department of Animal Science, College of Agriculture, Institute of Graduate Studies, Central Luzon State University, Science City of Muñoz, The Philippines

**Keywords:** *Aeromonas hydrophila*, *aer*A gene, minimum detectable limit, PCR assay

## Abstract

**Objective:**

The aerolysin (aerA) is a virulence indicator used to identify the pathogenicity of the *Aeromonas* strain. Targeting a pathogen’s crucial virulence gene for detection is essential, as it determines the potential threat to the host. This study aimed to develop a gold nanoparticle (AuNP) probe for detecting the gene *aer*A in *Aeromonas hydrophila* among field samples.

**Materials and Methods:**

Kidney samples among both healthy and sick Nile tilapias in five provinces of Luzon Island were collected for bacterial analysis. Screening using specific primers targeting *aer*A was conducted in parallel with testing the AuNPs probe on the same sample set. The positive control provided by BFAR-NFLD, confirmed by polymerase chain reaction (PCR) assay, was used as a positive sample containing the target gene.

**Results:**

The AuNP probe demonstrated a computed accuracy of 81.32%, sensitivity of 100%, and specificity of 81.26%. Among the 257 reactions, 59 were false positives, while no false negative results were observed. The AuNP probe could detect *aer*A at levels as low as 30 ng/µl. The low prevalence of the target gene may be attributed to the use of general media instead of specific media like Rimler-Shotts agar.

**Conclusion:**

The established colorimetric detection method for *A. hydrophila* with the *aer*A gene offers a swift alternative to PCR, negating the requirement for advanced equipment like a thermal cycler.

## Introduction

Gram-negative, rod-shaped bacteria that are facultative anaerobes, non-spore-forming, and belong to the genus *Aeromonads* are widely distributed in aquatic environments [1]. Aeromonads can be classified into two categories: psychrophiles, exemplified by *Aeromonas salmonicida*, and mesophiles, represented by *Aeromonas hydrophila*, which can infect both warm-blooded and cold-blooded organisms [2]. Sewage, sewage pollutants, surface water, groundwater, and sewage-contaminated waterways have all been shown to contain the *Aeromona*s species [3]. Due to their capacity to result in soft tissue infections, gastroenteritis, septicemia, and wounds to the host, they are clinically significant [4].

*Aeromonas hydrophila* was once considered an opportunistic pathogen connected to inferior bacteriological infections in freshwater aquaculture [5]. A virulent clonal population *A. hydrophila* was implicated in the establishment of motile *Aeromonas septicemia* (MAS) patients following an epidemic in 2009 in West Alabama and East Mississippi of farm-raised catfish MAS [6–6]. Market-sized catfish weighing tens of millions of pounds have been lost to virulent *A. hydrophila* [5,^6^], which has become a significant pathogen linked to catfish and carp farming in China [9] and the USA [10].

Countries including the United States, Egypt, India, and Spain [11-13], evidence concerning the reports, In-depth research has been done on polymerase chain reaction (PCR)-based virulence gene identification and detection of bacterial pathogens. Also, the application of nanotechnology for detecting these virulence genes has revolutionized their diagnostic capability.

Metal nanoparticles, with dimensions resembling those of biomolecules such as proteins (enzymes, antibodies), or DNA, share comparable sizes ranging from 2 to 20 nm. This similarity in dimensions fosters structural compatibility between these two material groups, aligning their structures effectively. Gold nanoparticles (AuNPs’) in many forms allow facile surface functionalization and operationalization with probes and other chemicals, making them accessible to various detection modalities [14].

AuNPs are currently popularly used in colorimetric biomolecule detection [15]. Due to their distinctive optical features resulting from surface plasmon resonance, which causes a substantial color change following the aggregation of AuNPs when they interact with different biomolecules, AuNPs are currently widely utilized for biomolecule detection [15]. In Thailand, researchers have established a visual identification method for the white spot syndrome virus by employing DNA-functionalized AuNPs as probes in conjunction with loop-mediated isothermal amplification [16]. Similarly, de Guia et al. [17] established an AuNP-based method for detecting acute hepatopancreatic necrosis disease (AHPND), specifically targeting the *pirA*vp toxin gene associated with the development of AHPND. Recently, Arunrut et al. [18] introduced a rapid and sensitive approach combining loop-mediated isothermal amplification with a DNA-functionalized AuNP probe for the colorimetric detection of the spore wall protein gene of the microsporidian *Enterocytozoon hepatopenaei*. This method relies on loop-mediated isothermal amplification and utilizes DNA-functionalized AuNPs as probes [18].

The detection of *A. hydrophila* using PCR and loop-mediated isothermal amplification (LAMP) assays was previously developed. However, both methods require incubation at least 65°C. The objective of this investigation was to create a probe utilizing AuNPs for identifying *A. hydrophila*. The development of such a protocol would alleviate the need for sophisticated equipment and shorten the detection time of economically significant diseases. With this, avoiding mass mortality on farms would be much easier.

## Materials and Methods

### Sample collection

Bacterial specimens were collected from both healthy and infected tilapia sourced from private farms in key tilapia-producing provinces: Pampanga, Isabela, Batangas, Laguna, and Nueva Ecija. Using a sterile cotton swab, samples were taken from the kidneys of the tilapia. These samples were then streaked onto sterile nutrient agar and incubated at 37°C for 24 h. After incubation, isolated colonies were selected and subjected to three successive sub-culturing steps to ensure the purity of the bacterial isolates. The colonies obtained from purified bacterial samples were utilized for DNA extraction through the boil method [19].

### Probe design and synthesis

The single-stranded DNA probe design was manually designed. Multiple sequences of the *aer*A gene from *A. hydrophila* were downloaded from the National Center for Biotechnology Information (NCBI). Alignment of sequences was carried out using Molecular Evolutionary Genetics Analysis version 7 to locate the conserved region of the *aer*A gene. Eighteen to 22 nucleotides were individually selected and then analyzed using Oligo Analyzer version 1.0.2 for hairpins and excessive secondary structures. The candidate probe, characterized by a guanine & cytosine (GC) content ranging from 40% to 60% and an annealing temperature within the range of 50°C–60°C, was assessed using Primer Blast to ensure it is devoid of any hairpin structure. Thiol C6 was placed on the 5’-end of the sequence to serve as the binding site for the AuNPs. The qualified probe sequence (5’-C6-TCA AGA CGG TGG TGG GCT GGG CGA T-3’) was sent for synthesis to Macrogen Inc. (South Korea).

### PCR assay

Initially, samples were screened for the presence of the *aer*A gene using PCR. The primers specifically designed for this study to target the *aer*A gene, consisting of the forward sequence (5’-TCAAGTGGCCACTGGTAGG-3’) and the reverse sequence (5’-AGGAAGCCACTCAGC GTC-3’), were utilized. The master mix, comprising 9.0 µl of ultrapure water, 6 µl of PCR buffer, 2 µl of MgCl_2_, 0.3 µl of deoxynucleotide triphosphates (dNTPs), 0.3 µl of each primer, 0.3 µl of Taq polymerase, and 2 µl of DNA template, was prepared. The temperature profile for the touchdown PCR involved an initial step at 95°C for 5 min, followed by 40 cycles of 95°C for 30 sec, 65°C with a 1.0°C decrease in temperature per cycle for 30 sec, and 72°C for 5 min. This was followed by one cycle at 72°C for 5 min.

### Synthesis and characterization of AuNPs

The AuNPs utilized in this study were produced using the Turkevich method [14]. UV-Vis spectrophotometry was used to analyze the AuNPs (ThermoFisher, USA), Fourier-transform infrared spectroscopy (FTIR) (ThermoFisher, USA), and scanning electron microscopy (SEM) (Hitachi High-Tech, Japan).

### Optimization of the colorimetric assay using AuNPs

Colorimetric detection was done by mixing 20 µl bacterial DNA plus the premix containing 10 µl of 1 M NaCl_2_, 10 µl 0.01M of phosphate buffer saline (PBS), and 10 µl probe. The mixture was left to incubate at ambient temperature for a duration of 1–2 min. For the verification of the result, 20 µl AuNP was added [20]. A change in color from reddish to grayish indicates positive results, while no change in color indicates negative results. The lowest detectable limit of *A. hydrophila* using the AuNP probe was determined. Using the equations presented by Baratloo et al. [21] accuracy, sensitivity, and specificity were calculated based on the results of PCR.

$Accuracy =\frac{TP+TN}{TP+TN+FP+FN}$

$Sensitivity =\frac{TP}{TP+FN}$

$Specificity =\frac{TN}{TN+FP}$

where:

TP = true positive;

TN = true negative;

FP = false positive; and

FN = false negative.

## Results and Discussion

The global proliferation of *A. hydrophila* poses a notable risk to aquaculture and the sustainability of fish production [22]. Addressing this challenge requires the creation of diagnostic techniques that are both sensitive and specific for detecting *A. hydrophila*. In this investigation, we examined the viability of utilizing an AuNP probe-based assay for the detection of *A. hydrophila*.

The AuNP probe demonstrated exceptional sensitivity, capable of detecting the target virulence gene at concentrations as low as 20 fg/µl [17]. This high sensitivity is a promising feature, as it allows for early detection of *A. hydrophila* infections, enabling prompt preventive and therapeutic measures to be implemented.

**Figure 1.**
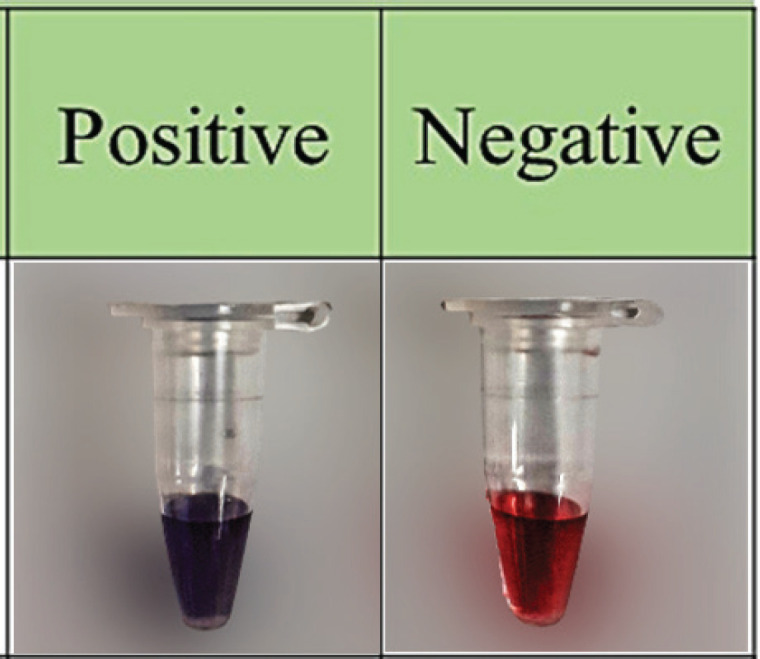
Colorimetric comparison of *aer*A gene positive (purple) and negative (red) samples.

The color change observed in the positive reaction of the assay is attributed to the attachment of the AuNP probe to the target complementary sequence (Fig. 1).

Moreover, the stability of the pinkish hue on AuNPs after bonding with the thiol group linked to the probe indicates a negative reaction [20]. This simple and visually interpretable colorimetric assay offers a rapid and user-friendly diagnostic tool for *A. hydrophila* detection, potentially suitable for on-site testing and point-of-care applications.

Furthermore, in a study conducted by Kampeera et al. [23], a modified oligoprobe labeled with AuNPs served as a colorimetric indicator, signaling the presence of specific LAMP products once the reaction concluded (after 45 min). The initially red colloidal AuNP solution underwent a transition to a grayish-blue hue upon the introduction of MgSO_4_ into the solution. This adjustment in AuNP coloration was fine-tuned to ensure a distinct differentiation between positive and negative outcomes, manifesting as red and grayish-blue hues, respectively, following the addition of salt. The impact of MgSO_4_ on probe hybridization and the ensuing color alteration was thoroughly assessed.

The 232-base pair amplicon generated in this process exhibited a 99% similarity to sequences of *A. hydrophila* documented on the NCBI platform, confirming its credibility as a virulence marker. The cytolytic toxin is encoded by the *aer*A gene and holds a crucial function in the pathogenicity of *A. hydrophila*. Upon proteolytic removal of its C-terminal propeptide, the membrane permeability barrier is broken, leading to the death of host cells [24]. Furthermore, *A. hydrophila* produces numerous virulence factors, such as cytotoxins, proteases, S-layers, and aerolysin, which contribute to the severity of infections [25]. Among the five tested virulence putative genes by Nhinh et al. [26] in different freshwater species, the *aer*A gene showed the highest frequency with 80.5%.

**Figure 2.**
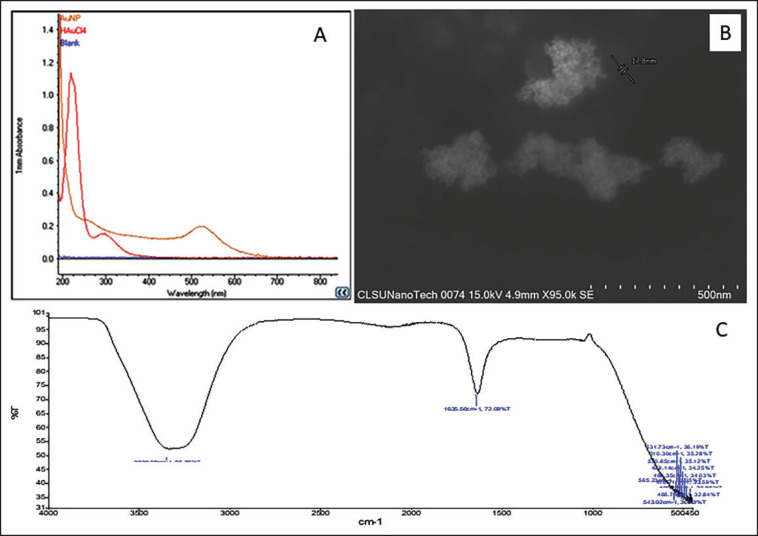
Characterization of AuNPs using UV-Vis (1A), SEM (1B) and FTIR (1C).

The successful preparation of 17.8 nm AuNPs is substantiated by characterizing them through FTIR, UV-Vis spectroscopy, and SEM, as illustrated in Figure 2.

These rigorous characterizations ensure the reproducibility and reliability of the AuNP probe-based assay, essential for its future applications as a diagnostic tool.

**Table 1.** Result summary of the AuNP probe testing.

Province	True positive (TP)	False positive (FP)	True negative (TN)	False negative (FN)	Total samples
Pampanga	0	9	48	0	48
Laguna	0	9	57	0	57
Batangas	0	14	47	0	47
Nueva Ecija	0	15	37	0	37
Isabela	1	12	67	0	68
Total	1	59	256	0	257

Table 1 presents a summary of the findings of the AuNP probe testing. The collected samples from 5 chosen provinces yielded a total of 257 bacterial isolates, specifically (Pampanga = 48, Laguna = 57, Batangas = 47, Nueva Ecija = 37, and Isabela = 68). Fifty-nine reactions from 257 samples are considered false positive results, with a maximum value of 15 from Nueva Ecija and a minimum value of 9 from Pampanga and Laguna. False-negative results were not observed in all of the samples tested.

Despite the high sensitivity observed in the AuNP probe-based assay, it is essential to acknowledge the limitation in terms of specificity, as the assay exhibited a specificity value of 81.26%, falling short of the standard value (95%) [22]. The observed low specificity could be attributed to potential cross-reactivity or non-specific interactions of the AuNP probe with other non-target DNA sequences. Further investigations are warranted to address this issue and improve the assay’s specificity. Modifications in the probe design, optimization of reaction conditions, and incorporation of additional control measures could potentially enhance the assay’s specificity, making it a more reliable diagnostic tool.

The AuNP probe-based assay showed promising results with its high sensitivity and successful detection of the *A. hydrophila* virulence gene. While it presents a valuable tool for *A. hydrophila* detection, addressing the specificity issue is crucial to ensuring its accuracy and reliability in diagnostic applications. The combination of PCR-based amplification and AuNP probes offers a powerful approach for sensitive and rapid detection of *A. hydrophila*, holding great potential for addressing the challenges posed by this pathogen in aquaculture settings [27].

## Conclusion

The computed values for the accuracy, sensitivity, and specificity of the AuNP probe were of good range. However, having only one positive sample is not conclusive as far as statistics are concerned. A better projection of the relevant parameters would call for more positive samples. The development of this AuNP probe could help in the rapid detection of *aer*A gene-possessing *A. hydrophila* and avoid mass mortality resulting in economic losses by utilizing early disease diagnosis.

## List of abbreviations

*aer*A gene, aerolysin gene; AuNPs, gold nanoparticles; BFAR-NFLD, Bureau of Fisheries and Natural Resources—National Fisheries Laboratory Division; dNTP, deoxynucleotide triphosphate; FTIR, Fourier-transform infrared spectroscopy; GC, guanine & cytosine; MAS, Motile *Aeromonas septicemia*; PBS, phosphate buffer saline; PCR, polymerase chain reaction; SEM, scanning electron microscope*.*

## References

[1] Haenen OLM, Dong HT, Hoai TD, Crumlish M, Karunasagar I, Barkham T (2023). Bacterial diseases of tilapia, their zoonotic potential and risk of antimicrobial resistance. Rev Aquac.

[2] Samayanpaulraj V, Sivaramapillai M, Palani SN, Govindaraj K, Velu V, Ramesh U. (2020). Identification and characterization of virulent *Aeromonas hydrophila* Ah17 from infected *Channa striata* in river Cauvery and *in vitro* evaluation of shrimp chitosan. Food Sci Nutr.

[3] Pendergraft MA, Belda-Ferre P, Petras D, Morris CK, Mitts BA, Aron AT (2023). Bacterial and chemical evidence of coastal water pollution from the Tijuana River in Sea Spray Aerosol. Environ Sci Technol.

[4] Semwal A, Kumar A, Kumar N. (2023). A review on pathogenicity of *Aeromonas hydrophila* and their mitigation through medicinal herbs in aquaculture. Heliyon.

[5] Bebak J, Garcia JC, Darwish A. (2012). Effect of copper sulfate on *Aeromonas hydrophila* infection in channel catfish fingerlings. N Am J Aquac.

[6] Hossain MJ, Sun D, McGarey DJ, Wrenn S, Alexander LM, Martino ME (2014). An Asian origin of virulent *Aeromonas hydrophila* responsible for disease epidemics in United States-farmed catfish. mBio.

[7] Tekedar HC, Waldbieser GC, Karsi A, Liles MR, Griffin MJ, Vamenta S (2013). Complete genome sequence of a channel catfish epidemic isolate, *Aeromonas hydrophila* strain ML09-119. Genome Announc.

[8] Zhang D, Pridgeon JW, Klesius PH. (2014). Vaccination of channel catfish with extracellular products of *Aeromonas hydrophila* provides protection against infection by the pathogen. Fish Shellfish Immunol.

[9] Zhang XJ, Yang WM, Li TT. (2013). The genetic diversity and virulence characteristics of *Aeromonas hydrophila* isolated from fishponds with disease outbreaks in Hubei province. Acta Hydrobiol Sin.

[10] Griffin MJ, Goodwin AE, Merry GE, Liles MR, Williams MA, Ware C (2013). Rapid quantitative detection of *Aeromonas hydrophila* strains associated with disease outbreaks in catfish aquaculture. J Vet Diagn Invest.

[11] González-Serrano CJ, Santos JA, García-López ML, Otero A. (2002). Virulence markers in *Aeromonas hydrophila* and *Aeromonas veronii* biovar *sobria* isolate from freshwater fish and from a diarrhoea case. J Appl Microbiol.

[12] Pridgeon JW, Klesius PH. (2011). Molecular identification and virulence of three *Aeromonas hydrophila* isolates cultured from infected channel catfish during a disease outbreak in west Alabama (USA) in 2009. Dis Aquat Org.

[13] Sarkar A, Saha M, Roy P. (2013). Detection of 232 bp virulence gene of pathogenic *Aeromonas hydrophila* through PCR based techniques: a rapid molecular diagnostic approach. Adv Microbiol.

[14] Aldewachi H, Chalati T, Woodroofe MN, Bricklebank N, Sharrack B, Gardiner PH. (2018). Gold nanoparticle-based colorimetric biosensors. Nanoscale.

[15] Shawky SM, Bald D, Azzazy HM. (2010). Direct detection of unamplified hepatitis C virus RNA using unmodified gold nanoparticles. Clin Biochem.

[16] Seetang-Nun Y, Jaroenram W, Sriurairatana S, Suebsing R, Kiatpathomchai W. (2013). Visual detection of white spot syndrome virus using DNA-functionalized gold nanoparticles as probes combined with loop-mediated isothermal amplification. Mol Cell Probes.

[17] de Guia ACM, Fernando SID, Medina NP, Eugenio PJG, Pilare RS, Velasco RR (2020). Gold nanoparticle-based detection of *pir*A^vp^ toxin gene causing acute hepatopancreatic necrosis disease (AHPND). SN Appl Sci.

[18] Arunrut N, Kampeera J, Sirithammajak S, Sanguanrut P, Proespraiwong P, Suebsing R (2016). Sensitive visual detection of AHPND bacteria using loop-mediated isothermal amplification combined with DNA-functionalized gold nanoparticles as probes. PLoS One.

[19] Rahman MM, Rahman MA, Monir MS, Haque ME, Siddique MP, Khasruzzaman AKM (2021). Isolation and molecular detection of *Streptococcus agalactiae* from popped eye disease of cultured tilapia and Vietnamese koi fishes in Bangladesh. J Adv Vet Anim Res.

[20] Ahmadi S, Kamaladini H, Haddadi F, Sharifmoghadam M R. (2018). Thiol-capped gold nanoparticle biosensors for rapid and sensitive visual colorimetric detection of *Klebsiella pneumoniae*. J Fluoresc.

[21] Baratloo A, Hosseini M, Negida A, El Ashal G. (2015). Part 1: simple definition and calculation of accuracy, sensitivity and specificity. Emerg (Tehran).

[22] World Organisation for Animal Health (2019). Aquatic animal health code.

[23] Kampeera J, Pasakon P, Karuwan C, Arunrut N, Sappat A, Sirithammajak S (2019). Point-of-care rapid detection of *Vibrio parahaemolyticus* in seafood using loop-mediated isothermal amplification and graphene-based screen-printed electrochemical sensor. Biosens Bioelectron.

[24] Kampeera J, Dangtip S, Suvannakad R, Khumwan P, Senapin S, Kiatpathomchai W. (2021). Reverse transcription loop-mediated isothermal amplification (RT-LAMP) combined with colorimetric gold nanoparticle (AuNP) probe assay for visual detection of tilapia lake virus (TiLV) in Nile and red hybrid tilapia. J Fish Dis.

[25] Verhamme IM, Leonard SE, Perkins RC. (2019). Proteases: pivot points in functional proteomics. Methods Mol Biol.

[26] Nhinh DT, Le DV, Van KV, Huong Giang NT, Dang LT, Hoai TD. (2021). Prevalence, virulence gene distribution and alarming the multidrug resistance of *Aeromonas hydrophila* associated with disease outbreaks in freshwater aquaculture. Antibiot.

[27] Nhinh DT, Le DV, Van KV, Huong Giang NT, Dang LT, Hoai TD. (2021). Prevalence, virulence gene distribution and alarming the multidrug resistance of *Aeromonas hydrophila* associated with disease outbreaks in freshwater aquaculture. Antibiotics.

